# Integrated enzyme reactor and high resolving chromatography in “sub-chip” dimensions for sensitive protein mass spectrometry

**DOI:** 10.1038/srep03511

**Published:** 2013-12-16

**Authors:** Hanne Kolsrud Hustoft, Ole Kristian Brandtzaeg, Magnus Rogeberg, Dorna Misaghian, Silje Bøen Torsetnes, Tyge Greibrokk, Léon Reubsaet, Steven Ray Wilson, Elsa Lundanes

**Affiliations:** 1 Department of Chemistry, University of Oslo, Post Box 1033 Blindern, NO-0315 Oslo, Norway; 2 School of Pharmacy, University of Oslo, Post Box 1068 Blindern, NO-0316 Oslo, Norway; 3 Department of Neurology, Akershus University Hospital, 1478 Lørenskog, Norway

## Abstract

Reliable, sensitive and automatable analytical methodology is of great value in e.g. cancer diagnostics. In this context, an on-line system for enzymatic cleavage of proteins, subsequent peptide separation by liquid chromatography (LC) with mass spectrometric detection has been developed using “sub-chip” columns (10–20 μm inner diameter, ID). The system could detect attomole amounts of isolated cancer biomarker progastrin-releasing peptide (ProGRP), in a more automatable fashion compared to previous methods. The workflow combines protein digestion using an 20 μm ID immobilized trypsin reactor with a polymeric layer of 2-hydroxyethyl methacrylate-vinyl azlactone (HEMA-VDM), desalting on a polystyrene-divinylbenzene (PS-DVB) monolithic trap column, and subsequent separation of resulting peptides on a 10 μm ID (PS-DVB) porous layer open tubular (PLOT) column. The high resolution of the PLOT columns was maintained in the on-line system, resulting in narrow chromatographic peaks of 3–5 seconds. The trypsin reactors provided repeatable performance and were compatible with long-term storage.

The determination of biomolecules in areas like cancer research or diagnostics are driven towards liquid chromatography (LC) combined with mass spectrometry (MS) due to their low abundance, need for selectivity and the limitation of sample sizes from various biospecimens^1^. Also, on-line sample preparation steps are currently being developed for less contamination and high recovery of small “precious” samples^2^. Regarding selectivity, the combination of LC and MS is considered a benchmark technology, as it can distinguish e.g. isoforms and subtle post-translational modifications. To improve sensitivity, miniaturized columns are used as the column inner diameter (ID) is inversely proportional with signal intensity when using a concentration sensitive detector like the electrospray MS^3^. The commercial nanoLC columns/chips systems (≈50–75 μm column ID) coupled to nanospray MS are robust and commonly implemented in routine analysis^4^,^5^.

To push sensitivity and thus detection limits even further, open tubular nanoLC columns with a stationary phase attached to the walls are arguably well suited, as the inner diameter of these columns is typically “sub-chip”, e.g. 10–20 μm. Karger and co-workers first investigated the use of such narrow ID porous layer open tubular (PLOT) columns for LC separations of peptides^6^,^7^,^8^. Rogeberg *et al*. later showed the separation of intact proteins on PS-DVB PLOT columns and found these columns to be highly efficient and giving low carry-over^9^. Such PLOT columns have lately been used for the sensitive identification of proteins from 1,000–10,000 cancer cells^10^,^11^.

A recent trend in bioanalysis is the development of proteomic platforms with on-line sample preparation^2^,^12^, with selective sample enrichment^13^, or for chemical reactions such as trypsin digestion^2^ to cleave proteins into easier detectable/identifiable peptides. Immobilized enzyme reactors (IMERs) have several advantages compared to in-solution based approaches, such as larger enzyme to substrate ratio, higher digestion efficiency, the possibility to be reused and to be used in on-line LC-MS platforms^14^. Numerous immobilized trypsin reactors have been developed with a handful commercially available, typically as particle packed or monolithic columns^15^,^16^,^17^. Recently, the combination of a porous layer inside a capillary with the enzyme bound to the porous surface was reported^18^,^19^.

In this study, we couple for the first time an enzymatic trypsin reactor on-line with LC-MS in open tubular, “sub-chip” dimensions. We use an enzyme reactor with trypsin immobilized onto a polymeric layer of 2- hydroxyethyl methacrylate- vinyl azlactone (HEMA-VDM)^20^, where the enzyme is attached using the azlactone functionalities of the VDM^21^. The HEMA provides hydrophilicity of the polymeric layer to avoid secondary interactions of biomolecules on the column^20^. The reactor (20 μm ID, 150 mm) was integrated in an on-line switching system together with a monolithic PS-DVB column (50 μm ID, 45 mm) for desalting and peptide enrichment, and a PS-DVB PLOT column (10 μm ID, 1 m) for peptide separation.

The system features high sensitivity and good repeatability with low carry-over. The low abundant protein progastrin-releasing peptide (ProGRP), a tumor marker for small cell lung cancer (SCLC), was used to evaluate the on-line system. Attomole levels of on-line digested ProGRP were detected in our automatable and easily operated nanoproteomic platform.

## Results

### Trypsin enzyme reactor design and characterization

**Figure 1:**
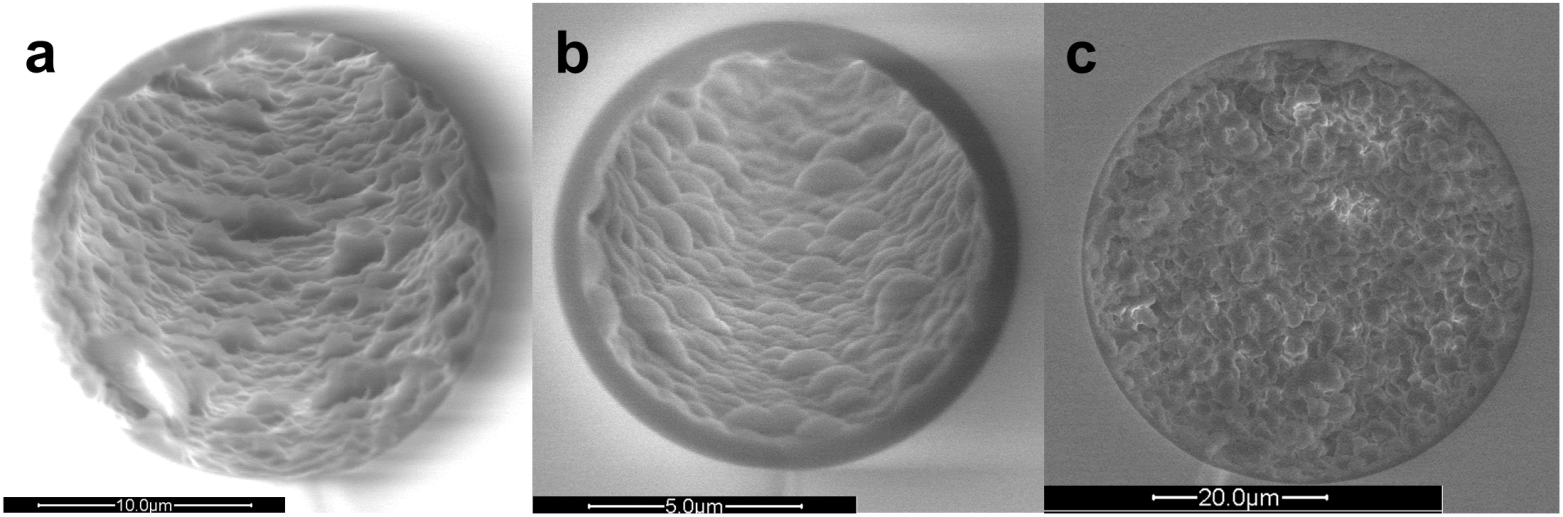
SEM images of the three capillary columns produced and used in the platform. (a): The inner polymeric layer of ≈ 0.75 μm (dried with nitrogen gas) of 20 μm ID HEMA-VDM reactor. (b): The 10 μm ID analytical PS-DVB PLOT column, with a ≈ 0.75 μm (dry) layer of polymer on the inside of the capillary. (c): The monolithic structure of the 50 μm PS-DVB solid phase extraction (SPE) column.

A 20 μm ID, 150 mm long polymeric open tubular trypsin reactor (made of HEMA and VDM monomers dissolved in the porogen 1-decanol) was produced through the reaction scheme shown in the methods section (see below). A scanning electron microscope (SEM) image of the trypsin reactor, with a polymeric layer of about 0.75 μm (dry) is shown in Fig. 1a. The reactor volume was 40 nL (18.5 μm ID, 150 mm length). Trypsin was bound to the polymeric layer through formation of a covalent link between the enzyme and the reactive functional groups of the VDM (as carried out by Krenkova *et al*^21^.). A column prepared without VDM did not show enzymatic ability, demonstrating the specific binding of trypsin and VDM.

### Trypsin reactor performance

The crafted trypsin reactors were tested for enzymatic cleavage with the SCLC biomarker ProGRP (isoform 1, 13.7 kDa)^22^, which is a key target analyte in our laboratory. In addition cytochrome C (11.7 kDa), a common protein standard for evaluating trypsin reactors, was used. Digestion time and temperature were set to 30 minutes and 37°C, allowing digestions to be performed during chromatography of the resulting peptides from a preceding enzyme cleavage. Peptides covering 54.1% of the aa sequence of ProGRP were detectable under these conditions (99% confidence, maximum 2 missed cleavages, see Supplementary Parameter Information 1), including its signature peptide (NLLGLIEAK, 485.8^2+^)^22^ (see Supplementary Fig. S1). In comparison, in-solution digestion of ProGRP resulted in detection of peptides covering up to 51.4% of the aa sequence (see Supplementary Fig. S2). For cytochrome C the sequence coverage obtainable was 88.6% (Supplementary Fig. S3) using the on-line platform (for chromatogram see Supplementary Fig. S4a). Maximum peak area of the signature peptide was obtained at 37°C, while only moderate changes in sensitivity and repeatability were observed with other reactor temperatures. Several identically made immobilized reactors were examined for operational stability and repeatability, and 5 out of 5 reactors (prepared individually) were able to perform on-line digestion and could provide detection of 5 ± 1 ProGRP peptides, all including the signature peptide (40.5–54.1% sequence coverage, see Supplementary Table S1 and Supplementary Fig. S1 and S5–S8). For comparison, a standard in-solution digest protocol allowed detection of 7 ± 2 ProGRP peptides (45.3–51.4% sequence coverage, see Supplementary Fig. S2 and S9–S10). Reactors were routinely replaced (due to simple reactor preparation and replacement), but possessed peak activity (≈50% sequence coverage of ProGRP) for at least 25 injections (see Supplementary Fig. S11–S14). However, the reactor required a few conditioning injections prior to obtaining maximal activity (e.g. ProGRP sequence coverage of injection #1 was 27.0%). Therefore, two blank/myoglobin injections, followed by digestion evaluation with cytochrome C were performed prior to ProGRP studies. The reactors showed excellent storage stability, being able to digest proteins after more than 4 months of storage in ammonium acetate, pH 6–7 at 4°C (Supplementary Fig. S3). Besides the standard cytochrome C and biomarker ProGRP, digestion of lysozyme (14.3 kDa) and myoglobin (17.7 kDa) was also successful.

### On-line trapping and separation in “sub-chip” dimensions

**Figure 2:**
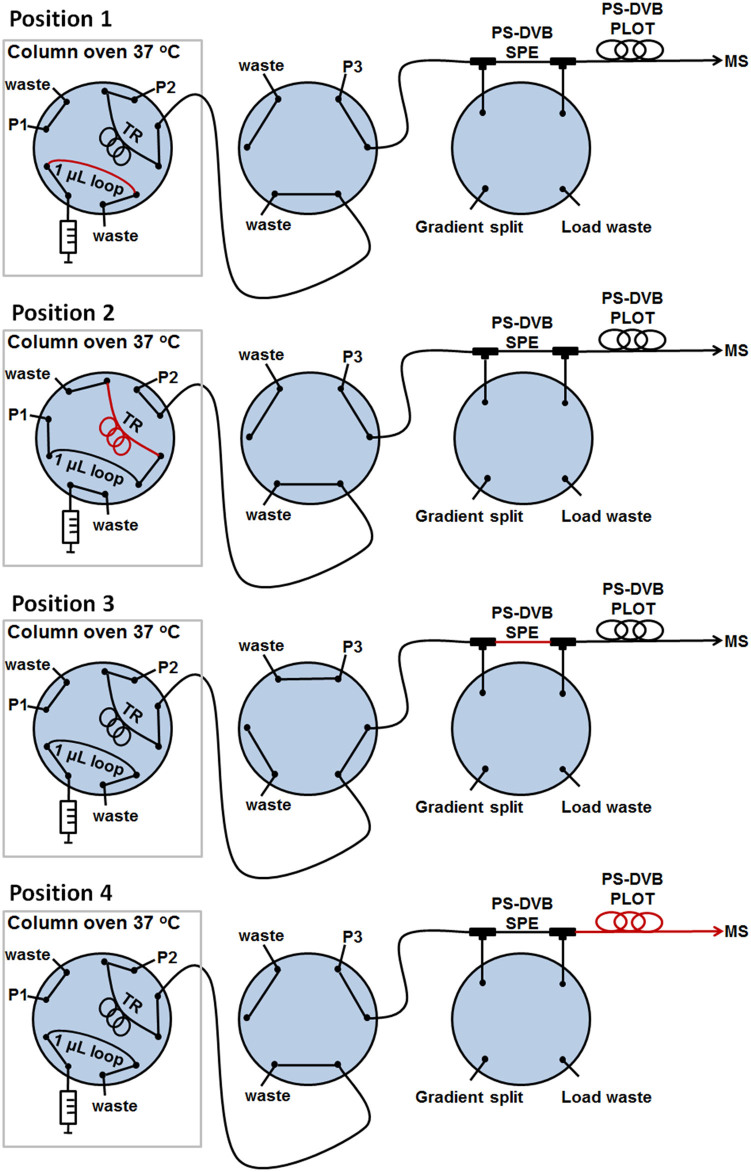
Experimental set-up of the nanoproteomic platform. Position 1: Sample injection. Position 2: Loading and subsequent digestion on trypsin reactor (TR). Position 3: SPE trapping. Position 4: PLOT LC separation with 40 nL/min flow. Washing and reconditioning of trypsin reactor. The red line represents the sample. See also the method section and animation (http://prezi.com/zxp2ioe_ecp2/lc-system/#) in the online version for more details.

The on-line system developed allowed peptides produced in the trypsin reactor to be transferred to a “sub-chip” PLOT LC column for chromatographic separation (see Fig. 1b for SEM image of the inside of the column). Fig. 2 presents a detailed outline of the system (see also animation of the nanoproteomic platform http://prezi.com/zxp2ioe_ecp2/lc-system/#). The system enabled proteins to be digested under ideal, slightly basic conditions, and for resultant peptides to be chromatographically separated and transferred to the MS under optimal, acidic conditions.

**Figure 3:**
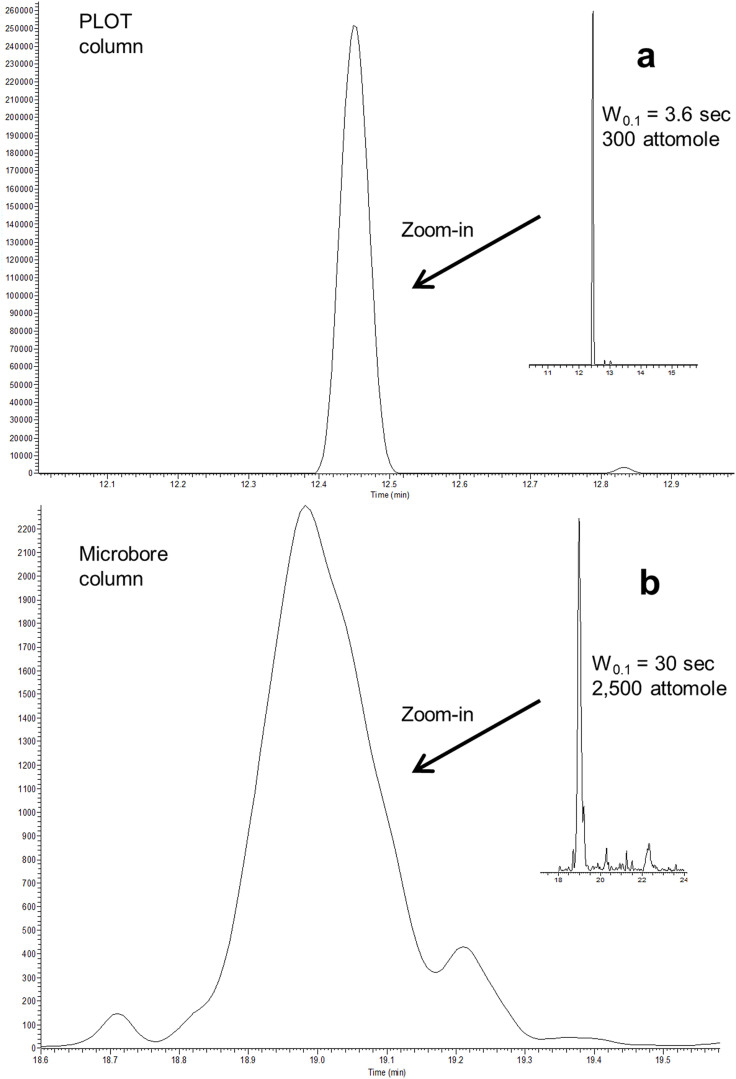
The signature peptide of ProGRP. Extracted ion chromatogram (EIC) of the signature peptide NLLGLIEAK (485.8^2+^). (a): W_0.1_ = 3.6 sec (485.8^2+^) from 300 attomole on-line digested ProGRP separated and detected in the novel nanoproteomic platform with PLOT peptide separation. (b): W_0.1_ = 30 sec (485.8^2+^) from 2,500 attomoles ProGRP, peptides separated on a conventional microbore column according to Torsetnes *et al*.^22^.

An isocratic pump 1 transferred the sample to the trypsin reactor, and the peptides produced were transferred and trapped onto a monolithic SPE column (which also served as a desalting step) using gradient pump 2 (see Fig. 1c for SEM image of the SPE column). Optimized condition for this step was using 4-minute trapping time with 4% acetonitrile (ACN) in 50 mM ammonium acetate, pH 8.75 (Supplementary Fig. S15). For the basic → acidic mobile phase replacement, the SPE with trapped peptides was flushed with 3.5 column volumes of mobile phase from pump 3 (0.1% formic acid in water) before a solvent gradient commenced, transferring the peptides from the SPE onto the PS-DVB PLOT column for chromatographic separation. The trypsin reactor was subsequently flushed with 50% ACN in 50 mM ammonium acetate (pH 8.75) and reconditioned with 4% ACN in ammonium acetate (pH 8.75), using pump 2, to be ready for a new digestion. Successful, low dead volume transfer of the analytes throughout the system was evident as chromatographic peak widths as low as 3.6 seconds (at W_0.1_ = 10% peak height) were observed (Fig. 3a). The SPE-PLOT system showed sufficient repeatability of retention time (relative standard deviation < 0.5%) evaluated with three peptides from cytochrome C (see Supplementary Table S2). The whole system held a pressure below 100 bar during the total cycle, enabling standard HPLC pumps to be used. Carry-over of ProGRP injected (e.g. 4–50 pg) was not observed. Coupled with a Q-Exactive Orbitrap mass spectrometer, our on-line trypsin reactor LC-MS system could cleave, chromatographically separate and detect down to 300 attomole of ProGRP unambiguously. One of the protein's signature peptides (evaluated using BLAST software^23^) was clearly present (see Fig. 3a) with a well-defined charge and mass fragment spectrum. For comparison a conventional packed column produced W_0.1_ peak widths of 30 seconds^22^ (Fig. 3b).

## Discussion

Hand-in-hand with demand for sensitive analysis of smaller samples in proteomic bioanalysis, miniaturization of LC systems has been pursued, as narrowing the column ID increases sensitivity when coupled with electrospray ionization mass spectrometry^24^,^25^. As conventional columns become exceedingly difficult to produce towards “sub-chip” sizes (i.e. low micrometer IDs), an alternative may be to use open tubular (OT) capillary LC columns. Historically, OT column applications in LC have been regarded as being merely theoretical, because of severe incompatibilities with instrumentation intended for conventional sized columns. This is to a large degree no longer the case, due to highly sensitive mass spectrometers and the development of PLOT LC columns and practical plumbing schemes^7^,^10^. Also, we can now assemble PLOT column based systems using only commercially available parts (with the exception of the easily prepared columns). The fact that narrow ID PLOT LC columns provide excellent performance in a multitude of set-ups (coupled on-line with solid phase extraction^10^, incorporated in a multidimensional LC^11^,^12^ and now coupled on-line with an enzymatic reactor (described in this paper)) is indeed a testament to their potential in modern bioanalysis. Although PLOT LC columns now provide excellent chromatography and sensitivity, we believe this will be augmented if the nanospray emitter is part of the column itself, as today's solution (coupling with larger emitters through a union) surely is a source of band broadening and non-optimal spray conditions. We are now developing such PLOT LC/emitter columns.

Parallel to the miniaturization of LC columns has been various attempts towards on-line sample preparation, for increased automation and minimizing sample loss, which is especially crucial regarding small sample sizes. In the case of on-line, immobilized enzymatic reactors (IMERs), an additional feature is elevated reaction rate compared to solution-based procedures^14^,^26^. IMERs have been the topic of a number of research and review articles^26^,^27^,^28^,^29^,^30^, various enzymes have been immobilized onto these reactors and used in flow systems^31^,^32^ with trypsin being by far the most common used IMER enzyme for cleaving peptides. Particles or monoliths are the most common supports for immobilized trypsin (with reactor size range of 4.6 mm down to 50 μm ID). Open tubular trypsin reactors (in sizes of 100–50 μm ID) have been produced and used for on-line digestion of minute amounts of protein^33^,^34^,^35^, though these earlier reactors suffered from a low available surface compared to monolithic reactors^18^. The combination of a polymeric layer inside the open tubular column increases the surface available to anchor the enzyme^18^,^19^, and favors the use of polymeric open tubular reactors with limited sample volumes.

In this paper, we have described the on-line coupling of a narrower (20 μm ID) polymeric open tubular trypsin reactor with PLOT LC. Using this ID together with a relatively short format, minute amounts of proteins were readily cleaved in a low backpressure reactor. A thorough optimization of the enzymatic reactor was performed; various porogens (e.g. ethanol, n-decanol) and monomer-to-porogen ratios were examined to create a surface enhancing polymeric layer on the column wall to anchor adequate amounts of trypsin. Most of the combinations resulted in clogged reactors, but a 1 + 6 ratio (HEMA/VDM+ n-decanol) gave a suitable polymerization solution for creating open polymeric layer reactors. The reactors were robust, enduring long-term storage, and variations in e.g. temperature were not overtly critical for performance. Although long term usage per reactor was not a goal (as we chose to replace reactors frequently), reactor activity did not diminish over 25 injections, which was shown to be the case in comparable studies^36^,^37^. Coupling with high resolving PLOT LC enhanced selectivity but also sensitivity, as peptides entered the MS in significantly more narrow bands compared to e.g. direct coupling of the trypsin reactor to MS (Supplementary Fig. S4).

Recently, members of our group have developed a protocol for detection and quantification of a very low abundant small cell lung cancer biomarker, ProGRP (reference limit levels of 58.9 pg/mL = 7.6 pM)^22^. Three isoforms of ProGRP were detected in the off-line antibody-based LC-MS method with estimated LOD of 500 attomole (3 × signal to noise ratio) using an SRM MS method^22^. Our prototype “sub-chip” reactor-LC-MS system could easily reach the detection limits of this previous method with significantly improved chromatography due to the use of PLOT LC (see Fig. 3a and b), providing the possibility for on-line, sensitive determination of small clinical sample amounts. However, to be able to handle the low pM samples analyzed by the off-line method, our “sub-chip” system will require a ProGRP affinity/enrichment column installed to enable analysis of microliters of sample. Kim *et al.* recently reported a similar immunoaffinity-enzyme reactor-separation system, using considerably larger dimensions^2^. Hence, our next step will be to develop a complete “sub-chip” system for targeted determination of ProGRP.

## Methods

### Preparation of PLOT and monolithic capillary columns

(For all abbreviations of chemicals and materials see Supplementary Chemicals and materials. For detailed procedure of making the columns and amounts of reagents, see animation, http://prezi.com/zxp2ioe_ecp2/lc-system/#).

For the pre-treatment 10, 20 and 50 μm ID and 360 μm outer diameter (OD) fused silica capillaries were filled with 1 M NaOH using an in-house made pressure bomb, the ends were plugged with GC septa and the capillaries were placed in an oven at 100°C for 2 h. The capillaries were washed with water and acetonitrile (ACN), followed by drying with nitrogen gas. A silanization solution containing 0.5% (wt./v) DPPH and 30% (v/v) 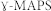 in DMF, was freshly prepared and the capillaries were filled with the silanization solution, the ends were plugged with GC septa and held in an oven at 110°C for 6 h. The capillaries were subsequently washed with ACN followed by drying with nitrogen gas.

#### SPE column

50 μm ID monolithic SPE columns were prepared by polymerization of 21% (wt. %) styrene with 19% (wt. %) DVB and 1% (with respect to monomer) initiator, AIBN. The binary porogenic mixture of 13% toluene and 47% 1-dodecanol (wt. %) was based on a preparation described by Lv *et al*.^38^. The polymerization temperature was 70°C with a duration time of 20 h.

#### PLOT column

The PLOT columns were prepared as described by Rogeberg *et al.*^9^. The column ID was 10 μm and the column length about 1 meter.

#### Trypsin reactor

**Figure 4:**
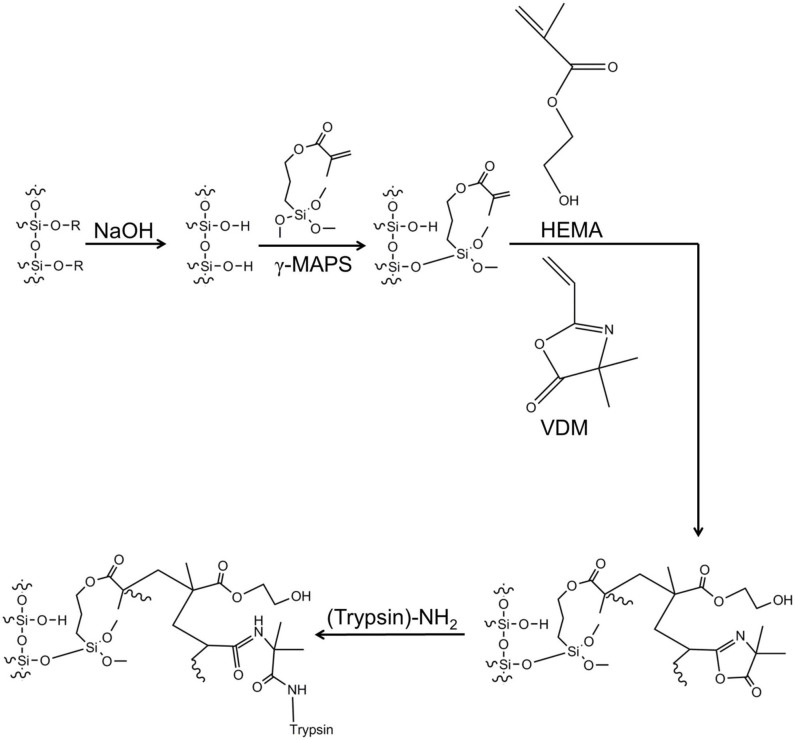
The reaction scheme of the HEMA-VDM PLOT trypsin reactor. See also Supplementary information and animation (http://prezi.com/zxp2ioe_ecp2/lc-system/#) in online version for more details.

Pre-treated capillaries were filled with a polymerization solution consisting of 80% (w/w) HEMA, 20% (w/w) VDM and 0.1% (w/w related to the monomer solution) AIBN in 1-decanol (weight ratio of monomer + porogen: 1 + 6) (Fig. 4). The capillaries were heated in two steps, first at 65°C for 5 h and then at 85°C for 5 h, and then dried with nitrogen gas for 1 h^20^. A 2.5 mg/mL trypsin solution was prepared by dissolving trypsin in 20 mM phosphate buffer, pH 7.4, containing 0.25 mg/mL benzamidine^21^. The porlymeric layer open capillaries were flushed with the trypsin solution for 3 h using nitrogen pressure (170–200 bar). Subsequently, the reactors were rinsed with 1 M ethanolamine in 20 mM phosphate buffer (pH 7.4) for 1 h to quench the unreacted azlactone functionalities of the VDM, followed by rinsing with ammonium acetate buffer (pH 6–7) and storage in ammonium acetate (pH 6–7) at 4°C.

### The nanoproteomic platform

Three pumps were used in the platform. Pump 1, an Agilent 1100 isocratic pump (Agilent, Sao Paulo, CA), was used for introducing the sample into the trypsin rector with a 0.5 μL/min flow for 1 min. The sample (added 50 mM tABC buffer and 5% ACN) was digested for 30 min at 37°C. The blank used between the runs had the same composition in order to monitor possible carry-over. Note that proteins containing disulfide bonds (lysozyme and myoglobin) was reduced and alkylated with a standard procedure^29^ prior to on-line digestion. For trapping of peptides generated in the trypsin reactor onto the SPE, a 1200 series Agilent pump (pump 2) with an Agilent G1379A series degasser was used. The flow was 0.5 μl/min, and peptides were trapped for 4 min. Pump 2 was additionally used for washing the trypsin reactor with 50% ACN and subsequent reconditioning with 4% ACN prior to the next digestion. For gradient peptide separation, an 1100 series Agilent pump (Pump 3) with a G1379A series degasser was used and the flow set to 2 μL/min. A 10-port VICI injector (Valco Instruments, Houston, TX) and two 6-port valves (valve 2 and 3) (Valco) were used in the platform. The 10-port valve was placed inside a column oven (Mistral, Spark Holland) at 37°C (25 and 45°C was also examined). Splitting of the flow from pump 3 was achieved by valve 2 with a ratio of 1:50, resulting in a PLOT column flow rate of 40 nL/min. The PLOT column was connected to a PicoTip™ nanospray tip (with 5 μm ID, at the tip) with a Picoclear™ union (both purchased from New Objective (Woburn, MA)). For pump 1 and 2, mobile phase A was comprised of 50 mM NH_4_OAc (pH 8.75) and mobile phase B comprised of ACN/NH_4_OAc (pH 8.75) (90%/10% (v/v)). 4% B was used for the trapping of peptides onto the SPE. For the gradient elution (pump 3) mobile phase A was comprised of H_2_O/FA (100%/0.1% (v/v)) and mobile phase B comprised of ACN/H_2_O/FA (90%/10%/0.1% (v/v/v)), and gradient elution with 5–40% B in 5 min and 40% B from 5 to 8 min was performed. For MS detection two different instruments were used MS 1 (most of the optimization of the reactor-LC system and Supplementary Fig. S1, S2 and S4–15 were performed this MS set-up): LTQ XL Orbitrap from Thermo (Thermo Fisher Scientific, Bremen, Germany) controlled by Xcalibur software (Thermo Fisher Scientific) was operated in positive ionization mode with CID fragmentation. Mass range was *m/z* 200–2000, and the electrospray voltage applied to the ESI needle was 1.3 kV. MS 2 (results shown in Fig. 3a and Supplementary Fig. S3, were obtained with this MS set-up): Q-Exactive Orbitrap (Thermo Fisher Scientific) was operated with Xcalibur software, positive ionization mode, HCD fragmentation, mass range *m/z* 200–2000 with 1.3 kV spray voltage on the needle. For data processing Thermo Proteome Discoverer 1.4.0.288 was used and the mass spectrometric raw data were database searched against their respective protein sequences in SEQUEST and Mascot. For the SEM procedure see Supplementary information, methods.

## Author Contributions

H.K.H., M.R., S.R.W. and E.L. planned and designed the experiments. D.M. performed preliminary experiments. H.K.H. and O.K.B. performed the experiments and data analysis. O.K.B. performed the SEM scanning. S.B.T. performed the experiment in figure 3b. H.K.H. wrote the manuscript. M.R., S.R.W., T.G., L.R. and E.L. reviewed and commented on the paper.

## Supplementary Material

Supplementary Information Supplementary file
