# Supplementary material for: Integrated enzyme reactor and high resolving chromatography in “sub-chip” dimensions for sensitive protein mass spectrometry
Source: Sci Rep. 2013 Dec 16;3:3511. doi: 10.1038/srep03511 (PMC3863811; doi:10.1038/srep03511)
Supplement: Supplementary Information — Supplementary file [file srep03511-s1.pdf]

Integrated enzyme reactor and high resolving chromatography in “sub-chip” dimensions for sensitive protein mass spectrometry

Hanne Kolsrud Hustoft[1,*], Ole Kristian Brandtzaeg[1], Magnus Rogeberg[1,3], Dorna Misaghian[1], Silje Bøen Torsetnes[2], Tyge Greibrokk[1], Léon Reubsaet[2], Steven Ray Wilson[1,*] and Elsa Lundanes[1]

[1]Department of Chemistry, University of Oslo, Post Box 1033 Blindern, NO-0315 Oslo, Norway, [2]School of Pharmacy, University of Oslo, Post Box 1068 Blindern, NO-0316 Oslo, Norway, [3]Department of Neurology, Akershus University Hospital, 1478 Lørenskog, Norway.

[*]Address correspondence to: h.k.hustoft@kjemi.uio.no and stevenw@kjemi.uio.no

Chemicals and materials

HPLC grade acetonitrile (ACN, VWR, West Chester, PA, USA), HPLC water (Chromasolv plus for HPLC, Sigma Aldrich, St. Louis, MO, USA), type 1 water from an ultrapure water purification system (Milipore Corporation, Billercia, MA, USA), formic acid (FA, 50 %, Fluka, by Sigma Aldrich), ammonium acetate (98 %, Sigma Aldrich), ammonia (28 %, VWR Fontenay-sous-Bois, France) were used to prepare the mobile phases. Myoglobin and lysozyme were reduced and alkylated prior to on-line digestion in the proteomic platform with Triethylammonium bicarbonate buffer (pH: 8.5±0.1) (tABC, Sigma-Aldrich), DL-Dithothreitol, (DTT, Fluka, Sigma Aldrich), Iodacetamide (IAM, Sigma Aldrich). The proteins used were cytochrome C (bovine heart, 1 mg/mL, 11.7 kDa), lysozyme (chicken egg white, 1 mg/mL, 14.3 kDa), myoglobin (equine heart, 1 mg/mL, 17 kDa. The proteins were all obtained from Sigma-Aldrich. Recombinant ProGRP isoform 1 was obtained as described by Torsetnes *et al.*[1]. To prepare the stock solution of the protein, 1 mg of the solid protein was dissolved in 1 mL of water to a 1mg/mL concentration. For preparation of the columns the following were used: N,N-Dimethylformamide anhydrous (DMF), 3-(Trimethoxysilyl)propyl methacrylate (γ-MAPS, 98 %), divinylbenzene (DVB, 80 % mixture of isomers, styrene, 99 %), 1-dodecanol, sodium hydroxide (NaOH, 99 %), inhibitor 2,2-diphenyl-1-picrythydrazyl hydrate (DPPH), ethylene dimethacrylate (EDMA, 98 %), 2-hydroxyethyl methacrylate (HEMA, 97 %, containing 200-220 ppm monomethyl ether hydroquinone as inhibitor) and initiator 2,2´ azobis(2-methylpropinonitrile) (AIBN), all purchased from Sigma Aldrich. Toluene was purchased from Rathburn Chemical Ltd. (Walkerburn, Scotland, UK). Trypsin from bovine pancreas (≥ 10,000 BAEE), benzamidine (>95 %), ethanolamine (99 %), sodium phosphate monobasic (99 %), di sodium hydrogen phosphate (99 %) and 1-decanol (98 %) were all purchased from Sigma Aldrich. The Vinyl azlactone (VDM) was purchased from Polysciences, Inc. (Warrington, PA). Ethanol was purchased from Arcus (Oslo, Norway). Nitrogen gas (99.99 %) was obtained from AGA (Oslo, Norway). Polyimide coated fused silica tubing (360 µm outer diameter (OD), 100, 75, 50, 20, 15, 10 and 5 µm ID) were purchased from Polymicro Technologies (Phoenix, AZ).

Methods

Scanning Electron Microscope (SEM) procedure

A sample of dried capillary (about 1 cm) was cut off, placed on a carbon tape, and placed in a FEI Quanta 200 FEG-ESEM (FEI, Hillsboro, OR, USA). The low vacuum mode was initiated while taking the SEM images and a LFD detector was used.

Tables

Supplementary Table S1: Trypsin reactor repeatability. Five reactors identically produced at different time points were able to perform on-line digestion and detect from ≈ 40.54-54.05% of the ProGRP aa sequence coverage (SQ), all digests including the signature peptide.

Reactor	Digest	% SQ	#Unique peptides
1	✔	54.05	6
2	✔	40.54	5
3	✔	40.54	5
4	✔	54.05	6
5	✔	40.54	4

Supplementary Table S2: Repeatability of retention time (RT): Three replicate run of cytochrome C. RT of three chosen peptides.

	Peptides *m/z*		
	792.88	728.83	1005.48
Replicate 1	13.68 min	13.90 min	18.16 min
Replicate 2	13.62 min	13.80 min	18.00 min
Replicate 3	13.68 min	13.87 min	18.08 min
Average	13.66 min	13.86 min	18.08 min
STDEV	0.03 min	0.05 min	0.08 min
**RSD %**	**0.25**	**0.37**	**0.44**

Figures

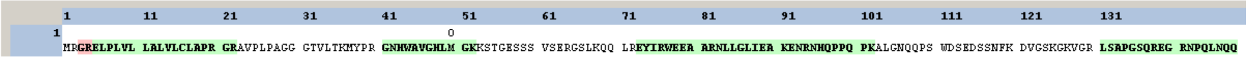

Supplementary Figure S1: Referring to Supplementary Table S1 reactor no #1. The sequence coverage from the new Protein Discoverer search (54.05 %, 6 unique peptides).

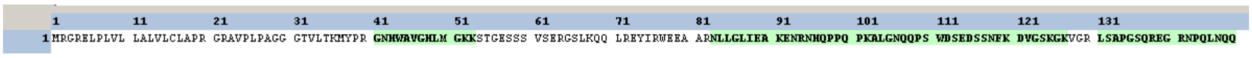

Supplementary Figure S2: Injection #2 of in-solution digested ProGRP isoform 1. The sequence coverage from the Protein Discoverer search (51.35 %, 9 unique peptides).

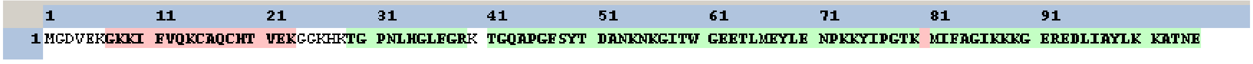

Supplementary Figure S3: Reactor prepared 25.09.2012: TOT2_040213_29: 3 µg/ ml cytochrome C, sequence coverage of 88.57 %.

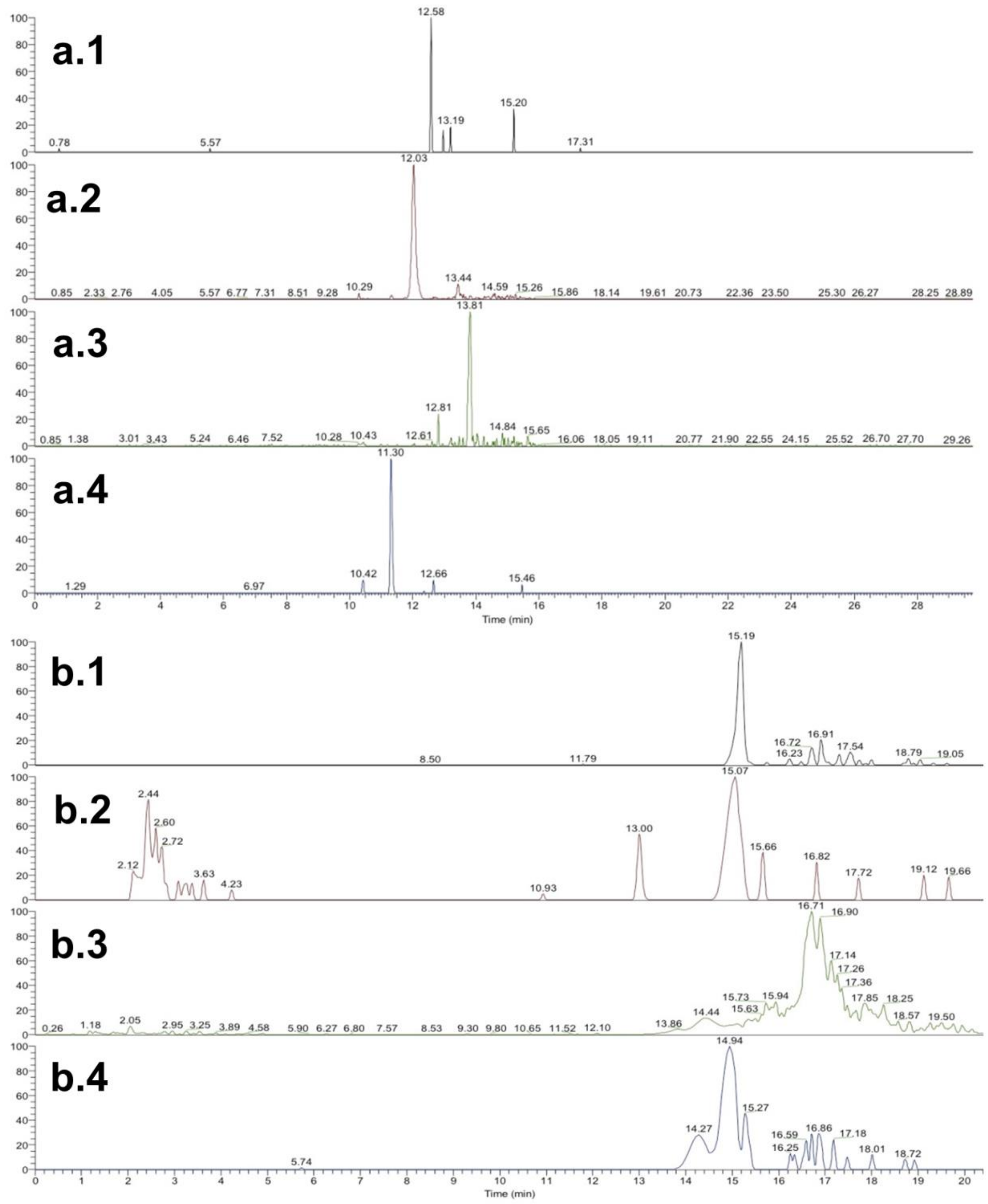

Supplementary Figure S4: PLOT separation gives high resolution: a: 5 µg/ml cytochrome C protein digested on-line and peptides separated on a PS-DVB PLOT column (40 nL/min flow). **b**: The same sample amount digested on-line with trypsin reactor and SPE directly coupled to the mass spectrometer (300 nL/min flow). EIC of peptides: 1. represents *m/z* 933.14^{3+}, 2. represents *m/z* 728.84^{2+}, 3. represents *m/z* 1013.48^{2+} and 4. represents *m/z* 1005.48^{2+}.

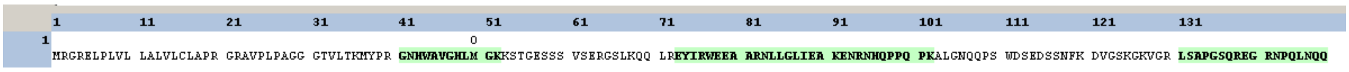

Supplementary Figure S5: Referring to Supplementary Table S1 reactor no #2. The sequence coverage from the new Protein Discoverer search (40.54 %, 5 unique peptides).

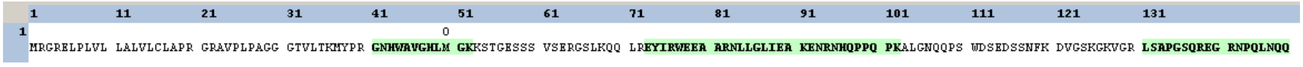

Supplementary Figure S6: Referring to Supplementary Table S1 reactor no #3. The sequence coverage from the new Protein Discoverer search (40.54 %, 5 unique peptides).

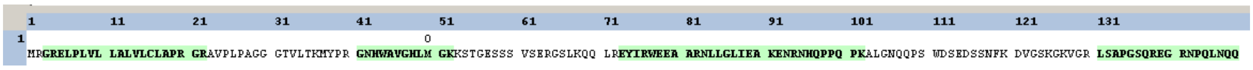

Supplementary Figure S7: Referring to Supplementary Table S1 reactor no #4. The sequence coverage from the new Protein Discoverer search (54.05 %, 6 unique peptides).

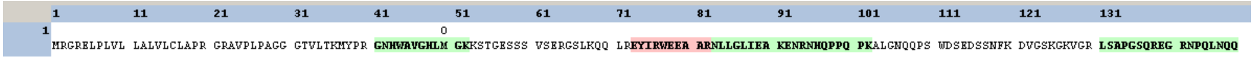

Supplementary Figure S8: Referring to Supplementary Table S1 reactor no #5. The sequence coverage from the new Protein Discoverer search (40.54 %, 4 unique peptides).

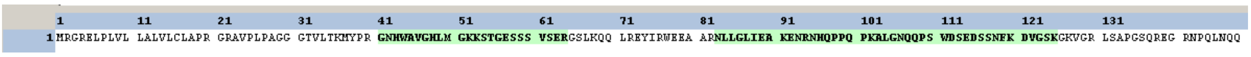

Supplementary Figure S9: Injection #1 of in-solution digested ProGRP isoform 1. The sequence coverage from the Protein Discoverer search (45.29 %, 5 unique peptides).

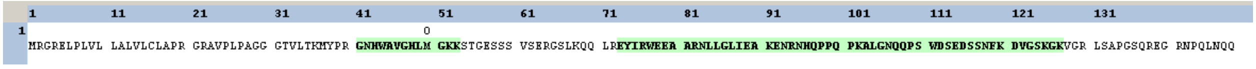

Supplementary Figure S10: Injection #3 of in-solution digested ProGRP isoform 1. The sequence coverage from the Protein Discoverer search (45.95 %, 7 unique peptides).

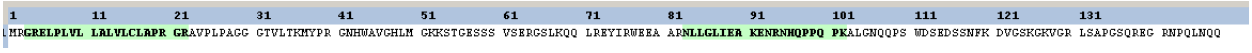

Supplementary Figure S11: Reactor #1 injection #1: 2/7-13 (ProGRP_250613_41): 10 µg/ml ProGRP, system without PLOT. The sequence coverage from the Protein Discoverer search (27.03 %, 4 unique peptides).

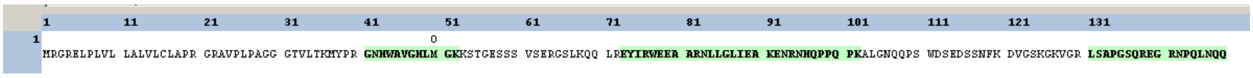

Supplementary Figure S12: Reactor #1 injection #10: 4/7-13 (ProGRP_250613_65): 5 µg/ml ProGRP, system without PLOT. The sequence coverage from the Protein Discoverer search (40.54 %, 5 unique peptides).

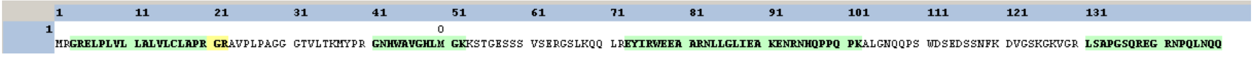

Supplementary Figure S13: Reactor #1 injection #15: 31/7-13 (TestFarm_300713_11): 5 µg/ml ProGRP, system without PLOT. The sequence coverage from the Protein Discoverer search (54.05 %, 7 unique peptides).

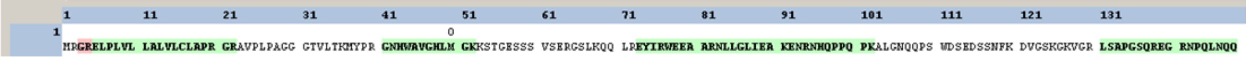

Supplementary Figure S14: Reactor #1 injection #25: 1/8-13 (TestFarm_300713_18): 5 µg/ml ProGRP, system with PLOT. The sequence coverage from the Protein Discoverer search (54.05 %, 6 unique peptides).

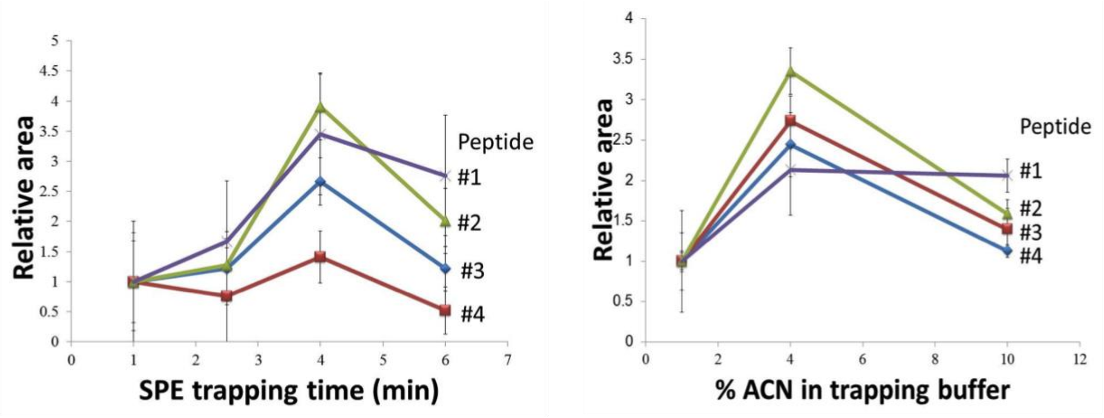

Supplementary Figure S15: Optimizing trapping time and organic content of trapping mobile phase. Left: Relative peptide area of 4 chosen peptides from in-solution digested myoglobin related to trapping time of the SPE. Right: Effect of relative area of the same peptides with percentage of organic content in the trapping buffer.

Parameter Information 1

Parameters from Proteome Discoverer Sequest HT search:

Created with Discoverer version: 1.4.0.288

==
======================

Number of filtered/unfiltered result items:
- 1/1 protein group(s)
- 1/1 merged protein(s)
- 8/8 peptide(s)
- 678/678 PSM(s)
- 6258/6258 search input(s)

==
======================

Peptide Grouping Options
- Show peptide groups: True
- Group peptides by: Mass and Sequence

Protein Grouping Options
- Enable protein grouping: True
- Consider leucine and isoleucine as equal: True
- Consider only PSMs with confidence at least: Medium
- Consider only PSMs with delta Cn better than: 0.15
- Apply strict maximum parsimony principle: True

No filters applied for data reduction

No result filters applied

==
======================

Summary of file D:\HKH\REVISED\TestFarm_300713_18.msf

Workflow created with Discoverer version: 1.4.0.288 (DBVersion:79)

==
======================

Search name: TestFarm_300713_18
Search description: -
Search date: 11/25/2013 09:51:53

==
======================

The pipeline tree:

```
  |-(0) Spectrum Files
    |-(1) Spectrum Selector
      |-(2) Sequest HT
        |-(3) Target Decoy PSM Validator
```

Processing node 0: Spectrum Files

Input Data:

File Name(s): D:\HKH\REVISED\TestFarm_300713_18.RAW

Processing node 1: Spectrum Selector
----

1. General Settings:

Precursor Selection: Use MS1 Precursor
Use New Precursor Reevaluation: True

2. Spectrum Properties Filter:

Lower RT Limit: 0.002405
Upper RT Limit: 25.0168866666667
First Scan: 0
Last Scan: 0
Lowest Charge State: 0
Highest Charge State: 0
Min. Precursor Mass: 200 Da
Max. Precursor Mass: 5000 Da
Total Intensity Threshold: 0
Minimum Peak Count: 1

3. Scan Event Filters:

MS Order: Is MS2
Activation Type: Is CID
Min. Collision Energy: 0
Max. Collision Energy: 1000
Scan Type: Is Full

4. Peak Filters:

S/N Threshold (FT-only): 1.5

5. Replacements for Unrecognized Properties:

Unrecognized Charge Replacements: Automatic
Unrecognized Mass Analyzer Replacements: ITMS
Unrecognized MS Order Replacements: MS2
Unrecognized Activation Type Replacements: CID
Unrecognized Polarity Replacements: +

6. Just for Testing:

Precursor Clipping Range Before: 2.5 Da
Precursor Clipping Range After: 5.5 Da

Processing node 2: Sequest HT
----

1. Input Data:

Protein Database: ProGRP_iso1.fasta
Enzyme Name: Trypsin (Full)
Max. Missed Cleavage Sites: 2
Min. Peptide Length: 6
Max. Peptide Length: 144

2. Scoring Options:

Max. Delta Cn: 0.05
Max. Number of Peptides Reported: 10

3. Tolerances:

Precursor Mass Tolerance: 10 ppm
Fragment Mass Tolerance: 0.6 Da
Use Average Precursor Mass: False
Use Average Fragment Mass: False

4. Spectrum Matching:

Use Neutral Loss a Ions: True
Use Neutral Loss b Ions: True
Use Neutral Loss y Ions: True
Use Flanking Ions: True
Weight of a Ions: 0
Weight of b Ions: 1
Weight of c Ions: 0
Weight of x Ions: 0
Weight of y Ions: 1
Weight of z Ions: 0

5. Dynamic Modifications:

Max. Equal Modifications Per Peptide: 3
Max. Dynamic Modifications Per Peptide: 4
1. Dynamic Modification: Oxidation / +15.995 Da (M)

Processing node 3: Target Decoy PSM Validator
----

1. Decoy Database Search:

Target FDR (Strict): 0.01
Target FDR (Relaxed): 0.05
